# View-Dependent Tessellation and Simulation of Ocean Surfaces

**DOI:** 10.1155/2014/979418

**Published:** 2014-02-06

**Authors:** Anna Puig-Centelles, Francisco Ramos, Oscar Ripolles, Miguel Chover, Mateu Sbert

**Affiliations:** ^1^Neuroscience Division, Starlab Barcelona, 08022 Barcelona, Spain; ^2^Department de Llenguatges i Sistemes Informatics, Universitat Jaume I, 12071 Castellon, Spain; ^3^Neuroelectrics, 08022 Barcelona, Spain; ^4^Department of Informatica i Matematica Aplicada, Universitat de Girona, 17071 Girona, Spain

## Abstract

Modeling and rendering realistic ocean scenes have been thoroughly investigated for many years. Its appearance has been studied and it is possible to find very detailed simulations where a high degree of realism is achieved. Nevertheless, among the solutions to ocean rendering, real-time management of the huge heightmaps that are necessary for rendering an ocean scene is still not solved. We propose a new technique for simulating the ocean surface on GPU. This technique is capable of offering view-dependent approximations of the mesh while maintaining coherence among the extracted approximations. This feature is very important as most solutions previously presented must retessellate from the initial mesh. Our solution is able to use the latest extracted approximation when refining or coarsening the mesh.

## 1. Introduction

Describing ocean waves is a very complicated challenge, as oceans are composed of different elements that form a very complex system. It is possible to find very complex mathematical models that simulate the behaviour of ocean waves, some of them are based on the direct observation of the sea [1, 2]. Nevertheless, the game industry usually prefers to lose physical realism due to the high demand for real-time simulation. Thus, real-time applications usually used simplified models that still offer physical realism but guarantee high frame rates.

Whitted was among the firsts to attempt the simulation of water [3]. In his simulation, the ripples were created by bump mapping the surface, perturbing the surface normal according to a single sinusoidal function, and ray tracing was used to obtain reflections.

**Figure 1:**
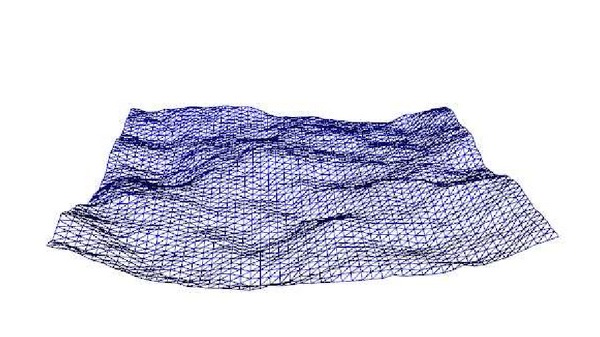
An ocean can be seen as an animated heightmap.

The approaches to simulate oceans that were based on bump mapping techniques [3, 4] cannot interact realistically with other surfaces or cast shadows on them. To avoid these shortcomings, Max [5] used a heightfield to render wave surfaces for his film “*Carla's Island*.” This approach is still followed and, therefore, oceans are usually simulated as unbounded water surfaces that are represented in the gaming environment as heightmaps. Other complex phenomena, such as foam, spray, or splashes, are usually modeled and rendered using particle systems [6–8]. In these simulations, the height of each vertex is modified in real time to offer the sensation of wave movement. It can be seen as the use of a displacement map to alter the position of each vertex [9].  Figure 1 depicts a snapshot of a mesh simulating ocean movement in a given instant of the animation.

Managing the geometry of the mesh representing the ocean still poses a limitation in simulating ocean. Kryachko [10] proposed the use of a static radial grid instead of a squared one. On that account, by centring this radial grid at the camera position we can have more points in those areas that are closer to the viewer. Although this solution is capable of offering more details in the areas closer to the viewer, it poses severe restrictions and does not assure a high performance. A more general technique that several authors propose is the tessellation of a squared heightmap. The tessellation process is capable of dividing a polygon in a set of smaller ones, thus enabling the application to alter the granularity of the initial mesh.

In this paper we propose a new ocean simulation using a GPU-based tessellation process. The main characteristics of the proposed ocean model are as follows.

- The ocean surface is refined on the GPU by means of a new view-dependent tessellation algorithm.
- Geometry shader capabilities are exploited to reuse extracted approximations.
- Wave movements are simulated with Perlin noise [4] on GPU.

The basic ideas of the solution presented in this paper were initially presented in a two-paged portfolio paper [11]. Due to the space restrictions, in that paper, the authors merely proposed the tessellation technique for ocean rendering. Thus, in the paper that we are now presenting, we thoroughly describe the tessellating and animating technique and we offer as well a complete study of the performance of our proposal.

This paper has the following structure.  Section 2 presents related work for oceans simulation and also for tessellation techniques.  Section 3 describes in detail the tessellation technique that we present.  Section 4 presents the oceans simulation process, which combines the tessellation technique with other processes to offer a realistic impression on GPU.  Section 5 presents the results obtained from a comprehensive study of the presented technique. Lastly, Section 6 concludes the developed techniques and offers future lines of work.

## 2. Related Work

In this state-of-the-art, we will firstly present the techniques that have been developed to offer a realistic visualization of the mesh simulating the ocean. We do not review here papers dedicated to running water or rivers such as [12], or the interaction of objects with ocean surfaces [13]. Nevertheless, a more general state-of-the-art report can be found in [14]. Later, we will describe the tessellation techniques that have been developed for ocean scenes.

### 2.1. Ocean Simulation

In this section we present a taxonomy of ocean simulation frameworks by following the type of animation of the oceans, as this is a key aspect for offering a realistic visualization. Following this classification we can distinguish between five sets of models for modeling ocean surfaces.

#### 2.1.1. Based on Parametrical Models

Parametric approaches represent the ocean surface as a sum of periodic functions which describe waves as a motion of particles. The physicist Gerstner presented the first theory in 1802 to approximate the solution to fluid dynamics by describing the surface in terms of motion of individual points on the surface [15]. Gerstner showed that the motion of each water particle is a circle of radius *r* around a fixed point, giving a wave profile that can be described by a mathematical function called trochoid.

One of the first descriptions of water waves in computer graphics was presented by Fournier and Reeves [16] using Gerstner waves. In the same year, Peachey proposed the generation of the heightfield by computing the superposition of several long-crested waveforms [17]. This author used particle systems to model the foam produced by wave breaking or colliding with obstacles. Later, [18] improved the wave simulation offered by the work of Fournier and Reeves. Tsó and Barsky [19] proposed a more precise way to solve the propagation (wave tracing) by approximating the resulting ocean surface with a Beta-spline surface, which the authors claimed to offer advantages over a polygonal representation. More recently, Cieutat et al. [20] extended the works based on fast Fourier transforms (FFTs) to manage correctly shore simulations. To sum up, we could say that all these approaches are very efficient although the scenes generated are not very realistic. We must note that noise is generally used in all the previous models to avoid visual regularity.

#### 2.1.2. Based on Physical Models

The Navier-Stokes equations offer a set of partial derivative equations which describe fluid movements. Kass and Miller [12] used simplified numerical methods to solve the Navier-Stokes equations for animation of water waves. Stam and Fiume [21] adopted FFTs to simulate the waves. More recently, Thürey et al. [22] proposed a simplification of the Navier-Stokes equations to offer real-time simulation of shallow water under some restrictions. Physical simulation approaches have a good quality of waves, but the implementation of these theories is usually difficult and simulating a large scene entails long computational times.

#### 2.1.3. Based on Spectral Models

This family of approaches, also known as statistical methods, is based on oceanographic measures, synthesized by spectral analysis. Spectral analysis assumes that the sea state can be considered as a combination or superposition of a large number of regular sinusoidal wave components with different frequencies, heights, and directions. As an example of these oceanographic measures, in 1964 Pierson and Moskowitz [23] developed a model for the spectrum of fully developed wind seas on 460 ship-recorded waves.

Mathematically, spectral analysis is based on the Fourier transform of the sea surface. Hence, these methods represent the ocean surface as a heightfield computed from a sum of sinusoids of various amplitudes and phases; small-scale waves and ripples are modeled directly by adding noise perturbation [24, 25]. Spectral solutions were firstly introduced by Mastin et al. [1]. The basic idea is to produce a heightfield having the same spectrum as the ocean surface. The main benefits of this approach were that many different waves are simultaneously simulated, with visually pleasing results. Premoze and Ashikhmin [26] combined physical models and oceanography models, but the obtained solution was only adequate for calm sea.

Tessendorf [25] showed that dispersive propagation can be managed and that the resulting field can be modified to yield trochoid waves. More recently, Mitchell from ATI [27] introduced a Fourier-based GPU-synthesized height and normal maps. From a different perspective, Gonzato et al. [2] proposed a semiautomatic method to reconstruct the surface of the ocean from a video containing a real ocean scene. Finally, Nielsen et al. [28] proposed recently a method to allow artists to quickly sketch the waves appearance and automatically approximate and animate them.

Summarizing, these approaches ensure high realism, but they are not easily controllable. Moreover, since the mathematic model and the related computations are very complex, these methods are more adequate for animation than for real-time rendering.

#### 2.1.4. Based on Time-Varying Fractals

Fractals can be an adequate solution for simulating open sea, although they would not be capable of simulating how waves break on the seashore. A very general procedural technique for the simulation of water surfaces by means of stochastic fractals was proposed in [29]. Perlin [4] used a noise synthesis approach to simulate the appearance of the ocean surface seen from distance. It could be considered as a particular kind of stochastic fractal that is generated as an addition of several copies of a continuous noise function. Johanson [30] adopted this approach to simulate a small ocean surface. In paper [31], the authors showed that vertex shaders can be exploited to interactively generate nonstationary stochastic fractals to simulate the dynamics of water. Later, in Yang et al. [32], the authors used Perlin noise to generate the heightfield of an unbounded ocean surface. Although it has been shown that this particular kind of simulation is only well suited for a limited kind of wave phenomena, its ease, efficiency in implementation, and the possibility to use this process to simulate other phenomena make it a very appealing alternative.

#### 2.1.5. Hybrid Approaches

To overcome the problems of each family of solutions, hybrid procedural models were proposed. Thon and Ghazanfarpour [33] used a hybrid approach where the spectrum synthesized using a spectral approach was used to control the trochoids. This was only applicable in the calm sea case. Fréchot [34] presented a new hybrid approach where the effort was focused on wave animation and not in other effects like Fresnel reflectivity or foam. The authors used classical oceanographic parametric wave spectra to fit real-world measurements, applying Gerstner parametric equations and Fourier transform. More recently, Darles et al. [35] integrated a wave model defined as an amount of trochoids waves into a unique data structure. This data structure allowed them to consider spatial and temporal coherence as well as reducing aliasing effects.

### 2.2. Tessellation Techniques for Ocean Rendering

Terrain tessellation has been researched for a long time (see [36] for a complete survey), and many of the developed techniques can be applied to ocean simulation. Nevertheless, there have been specific attempts to generate real-time ocean surfaces on graphics hardware. Some authors have developed specific solutions to generate real-time ocean surfaces on graphics hardware. Schneider and Westermann [31] entirely performed visual simulation on the GPU at interactive rates. They used OpenGL evaluators and NURB surfaces to tessellate the geometry on GPU. Moreover, they also used vertex shaders to generate the noise function that animates water simulation. Presenting a simple LOD management, the work described in [37] offered a solution where the wave geometry is represented as a dynamic displacement map for close areas (near patch) and a dynamic bump map for farther areas (far patch). The nearest patch could change its resolution according to the height of the viewpoint, while the far patch is precalculated and relocated during simulation. They used the spectral method of Tessendorf [25] to animate the ocean surface. Later, Cieutat et al. [20] proposed a view-dependent level-of-detail solution where cracks are avoided thanks to the use of a textured plan placed under the sea surface.

Recently, adaptive schemes have successfully been used for efficient modeling, rendering, or animation of complex objects. The idea is to minimize the sampling of the geometry according to criteria such as the distance from the viewpoint. Since the adaptive sampling is done on the fly for each frame, this fits well with procedural surface displacement, which can easily be animated. Hinsinger et al. [38] relied on an adaptive sampling of the ocean surface, dictated by the camera position. Moreover, their animation model was also adaptive, since they filtered the waves that cannot be observed from the current viewpoint. The tessellation and waveform superposition were performed on the CPU and uploaded to the GPU each frame, which was the bottleneck of their approach. Later, Johanson [30] presented the projected grid concept, where the vertices of a grid were even spaced in postperspective camera space. The authors described how to develop a fully GPU implementation, although it was not performed.

In paper [32], the authors offered adaptive GPU-based ocean surface tessellation by using a previous adaptive scheme for terrain rendering. Their tessellation scheme avoided the loading of vertex attributes from CPU to GPU at each frame. Their main limitation was the fact that their tessellation scheme used a restricted quad-tree where two neighbouring areas with different resolutions could only vary to a limited extent. Also, in [39], authors presented an ocean simulation which was adaptively tessellated and driven by both per-vertex waves and per-pixel waves, using the Gerstner wave model for animating the ocean due to its simplicity and nonperiodicity. The tessellation occurred in eye space, mapping a regular grid to the intersection of the ocean plane and the camera viewport. This allowed them to only simulate and render geometry that is seen and tessellates more finely in the foreground than in the background. Lastly, Chiu and Chang [40] offered an adaptive GPU-based ocean surface tessellation, where the refinement took place in screen space. Moreover, they also provided optical effects for shallow water and spray dynamics by means of particle systems.

### 2.3. Characterization of Ocean Models

Table 1 presents a summary of a comparison of the most recent methods from those that have been presented in this section. Among the columns that this Table presents, it is worth mentioning that the column labeled as Others includes different additional features that can be considered in the different models. These additional features mainly refer to physical properties like the Kelvin wedge, which refers to the specific pattern of waves produced by moving ships on open water [41] or optical effects like the bidirectional reflectance distribution function (BRDF), which considers how light is reflected at a surface depending on its properties and on the camera position.

## 3. Our GPU-Based Tessellation Scheme

As we have mentioned in the previous section, tessellation is a widely used technique in ocean simulation. Adaptive approaches are much more interesting, as they can refine those areas that need more details, while those areas which are less interesting can be coarsened. Nevertheless, there is no ocean tessellation technique which considers the use of the latest features of graphics hardware. It is our objective to exploit these features in order to improve the performance of previous adaptive tessellation techniques.

**Figure 2:**
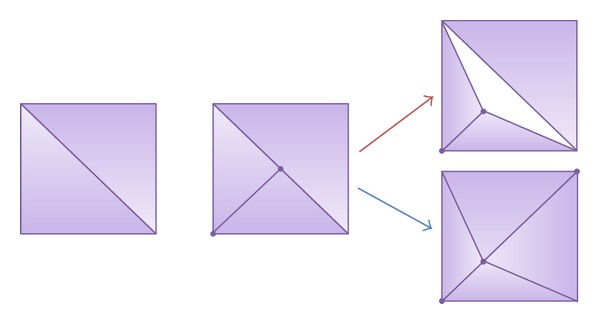
Example of crack after a tessellation step. Correct tessellation to avoid crack is also shown.

Many of the tessellation algorithms presented in the state-of-the-art section modify the details of the triangles following some criteria applied to the triangle. The calculations involved could consider the distance of the triangle to the camera or its position on screen. Nevertheless, applying the level-of-detail criterion in a triangle basis implies a limitation for adaptive solutions. As an example, Figure 2 presents a tessellation step where the bottom-left triangle has to be refined, while its neighbour does not have to. Later, if we apply some modifications to the position of the vertices we can obtain a noticeable crack, a hole in the mesh, as shown in the top-right image. These cracks are due to the introduction of T-vertices in the input mesh. T-vertices appear commonly in tessellation algorithms when a vertex is positioned on the edge of another triangle [42], resulting in two edge junctions making a T-shape. An example of this problem can be seen in Figure 2, where the vertex added in the tessellation step represents a T-vertex.

In order to avoid crack problems, some authors apply the refinement criterion only to the edges of the triangle. Therefore, if an edge needed refinement, then both triangles sharing the edge would act accordingly. In this case, following the example presented before in Figure 2, both adjacent triangles would perform the appropriate tessellation tasks to create new triangles with the same new vertices, assuring that no crack is generated (see the bottom-left image in Figure 2).

### 3.1. Tessellation Patterns

**Figure 3:**
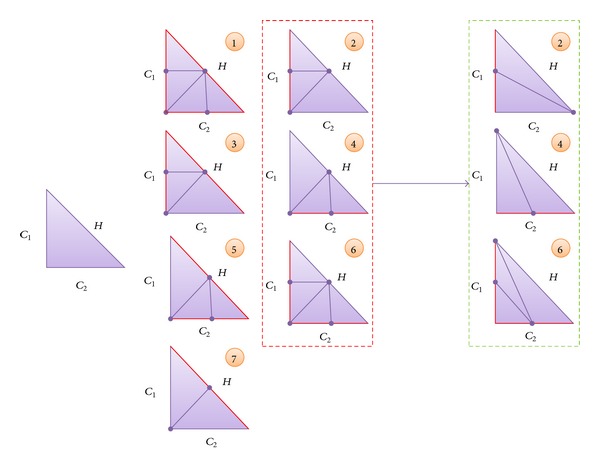
Tessellation patterns from Ulrich [43], where the red colour indicates the edges that need refinement. Patterns surrounded by a red-dotted line include T-vertices, while those surrounded by a green-dotted line offer tessellations without T-vertices [44].

Guided by the idea of developing an edge-based tessellation algorithm that avoids cracks, Ulrich described some edge-based patterns for tessellating triangles [43].  Figure 3 presents, on the left side, an initial rectangular triangle where its hypotenuse and catheti (more commonly known as legs) are depicted anticlockwise as *H*, *C*_1_, and *C*_2_. Next, the seven tessellation patterns introduced by Ulrich are presented (labeled from 1 to 7), where the edges of the original triangle that need refinement are in red. As we stated before, the work that we are proposing is based on using a refinement criterion based on the edges and not on the complete triangle. As a result, each pattern shows the tessellation that would be necessary depending on the combination of edges that need refinement. For example, in the bottom-left case the hypotenuse needed refinement and a new vertex has been added to create two new triangles. The main problem with Ulrich's proposal was that some of his patterns were based on the use of T-vertices (those surrounded by a red-dotted line in Figure 3). To avoid cracks, Ulrich proposed propagating the tessellation to neighboring triangles. Nevertheless, this propagation is not necessarily limited to a local neighborhood and, thus, his scheme is difficult to parallelize on the GPU.

In order to avoid this limitation, the work presented in [44] modified the previous patterns that included T-vertices. In Figure 3 the three modifications for patterns 2, 4, and 6 are shown surrounded by a green-dotted line, where it can be seen how no T-vertex is added.

In our case, we will use the patterns presented in [44], as they can assure that the continuity of the mesh is maintained without resorting to complex neighborhood analysis. These patterns produce more elongated triangles if compared with Ulrich's patterns, which could result in more complex lighting or texturizing. Nevertheless, our algorithm will calculate these values from the vertices of its parent triangle.

### 3.2. Our Proposed Algorithm

As we are processing the mesh in a geometry shader, each triangle is processed separately. For this reason, we have developed a technique which can alter the geometry of two triangles that share an edge without any communication among them. With this approach we will be able to exploit the parallelism of graphics hardware.  Algorithm 1 offers some pseudocode of the main tessellation process which is performed in the geometry shader unit.

#### 3.2.1. Adding Details to the Mesh

When refining the mesh, the algorithm checks the edges of each triangle to see whether they need refinement. Depending on the edges that need more detail, the algorithm selects a pattern for tessellating the input triangle (see Figure 3). Each of these generated triangles stores the spatial coordinates, the texture information, and any other information needed for rendering. Moreover, it is necessary to output for each new triangle two pieces of information that enable our solution a number that uniquely identifies the triangle and a number that codes the patterns applied.

The identification value *I* and the patterns information *P* of the triangles generated at each tessellation step can be calculated using (1) and (2), respectively, where

(1) $$\begin{array}{c} I=I*\delta +\gamma +c, \end{array}$$

(2) $$\begin{array}{c} P=P*\alpha +p \end{array}$$

- *δ* refers to the maximum number of triangles that can be output from all the tessellation patterns;
- *γ* is the initial number of triangles of the ocean mesh;
- *c* is a value in the range [0, *δ* − 1] which enables the tessellation algorithm to assign a different identification value to each triangle belonging to the same parent. Thus, when tessellating a triangle, each child will be assigned a different *c* value and, therefore, will have a different *I* value;
- *α* refers to the number of different patterns available;
- *p* is a value in the range [1, *α*], as it indicates the pattern that was applied when tessellating the current triangle.

As we can see, each *I* value will be different for each triangle, while all the triangles belonging to the same parent will have the same *P* value. These two values are the elements that enable our algorithm to recover less detailed approximations without having to start again from the coarsest approximation. It is important to underline that this is one of the main features of the method that we propose.

**Figure 4:**
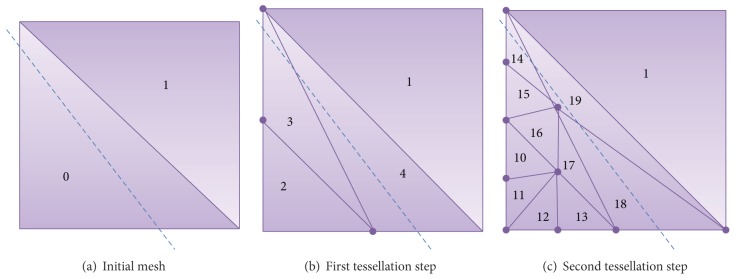
Tessellation example with the *I* value of each triangle.

In Figure 4 we present an example of this process. In this example, the *δ* value is equal to 4 as this is the number of triangles that are generated when all the edges need refinement (see pattern number 1 in Figure 3), while *α* is equal to 7 as this is the number of patterns available (see Figure 3). The *γ* value is equal to 2, as the initial mesh of our example (see Figure 4(a)) is composed of two triangles. Note that, initially, the *I* values of the triangles are given sequentially (starting from 0) and all the *P* values are equal to 0. The dotted line in blue of these figures divides the mesh in two areas, so that the area below the line is supposed to need refining.

Following on with this example, each of the two initial triangles go through the extraction process of the algorithm that we are presenting. In the specific case of the triangle number 1, the algorithm detects that none of its edges needs refinement and, as a consequence, no change will be made. Nevertheless, the algorithm detects that triangle with I 0 needs refinement because the center points of the two legs of the triangle are below the dotted line. Then, we choose from the patterns the one that reflects this combination and we apply it, so that we obtain the three new triangles shown in Figure 4(b). It can be seen how the *I* values of the new triangles are calculated following the formula (1), assuring that no repeated *I* is given. Following with the refinement process, the next tessellation step shows that different patterns have been applied to triangles 2, 3, and 4, as they represent different types of tessellation.

**Figure 5:**
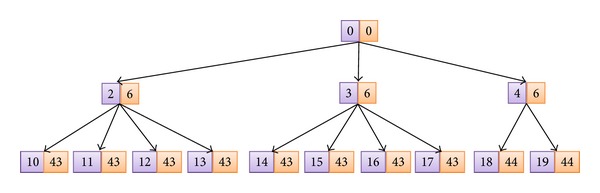
Tessellation tree. Each node presents the *I* and the *P* values of each triangle.

Figure 5 presents the tree of triangles that can be obtained in the example that we are presenting. For each node we present, on the left and in blue color, the *I* of the triangles and on the right and in red color the *P* value of each triangle. Both sets of values are calculated following the formulas presented in (1) and (2). It is important to mention that the number of children of each node will depend on the pattern applied, as they output a different number of triangles. By using the previously proposed tessellation patterns in Figure 3, we can refine one triangle and obtain 2, 3, or 4 new triangles.

#### 3.2.2. Removing Detail from the Mesh

Our proposed tessellation algorithm has been devised to offer a very efficient coarsening of the surface mesh, as we can reuse the latest approximation without having to start from the initial mesh. Thus, following with the previous example, if we wanted to reduce the detail and return to the state shown in Figure 4(b), each of the triangles located under the dotted line would execute the same coarsening process. The basic idea is that, for all the triangles belonging to the same parent, only one should be kept and its coordinates should be modified to recover the parent.

The *I* and *P* values of the mesh enable us to return to the previous tessellation status by means of the following equations:

(3) $$\begin{array}{c} c=\mathrm{mod}\left(\left(I-\gamma \right),\delta \right), \end{array}$$

(4) $$\begin{array}{c} p=\mathrm{mod}\left(p,\alpha \right), \end{array}$$

(5) $$\begin{array}{c} I=\frac{I-\gamma }{\delta }, \end{array}$$

(6) $$\begin{array}{c} P=\frac{P}{\alpha }. \end{array}$$

We must remember that, in the refining process, each triangle generated from the same parent had a different *I* value thanks to the *c* value. For coarsening the triangles, this value will be useful to differentiate between the child triangles and decide which one should be kept. More precisely, in those cases where this value is equal to 0, the algorithm assumes that this triangle is in charge of recovering the geometry of the parent triangle.

Once we know which triangle is responsible for becoming the parent triangle, we must know which tessellation pattern was used to generate it. In this case, the *p* value can be calculated with (4) so that we can know which pattern was applied and how to modify the coordinates of the vertices.

At this point, the only task that remains is to calculate the new *I* and *P* values using the appropriate equations. In this sense, the triangle that has been chosen to recover the parent will have its coordinates and the same set of *I* and *P* values. In this way, we could continue coarsening the mesh without obtaining any crack or artifact as our algorithm can process the triangles in an independent manner.

Following on with the example presented in Figure 4, if we wanted to coarsen the geometry each triangle would go through a coarsening process. Let us suppose that we are processing the triangle with I 10. If we calculate its *c* value, we obtain a 0 value, indicating that triangle with *I* equal to 10 is the one that must become the parent triangle, whose *I* can be retrieved with (4). In this case, the *p* would indicate that pattern 1 was applied and we would calculate the spatial coordinates of the parent triangle accordingly. Nevertheless, as triangles 11, 12, and 13 have a *c* different from 0, they would be discarded.

### 3.3. Camera Movement

In the previous section we have described the tessellation process but we have considered that the conditions used to decide which triangles to tessellate are not modified. In this way, in the example presented above (see Figure 4), we have considered that the location of the plane remains unaltered. Nevertheless, in a real case, the conditions of the criterion that guides the tessellation algorithm are modified continuously as they are usually related to the camera position.

**Figure 6:**
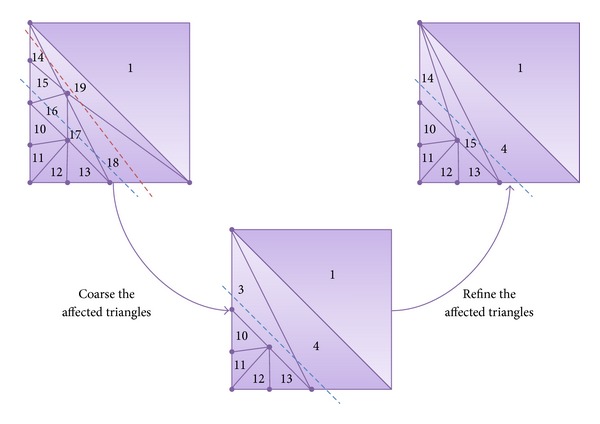
Example of retessellation when the refinement criterion is changed (old criterion in red and the new in blue).

Figure 6 is based on the second tessellation step shown in Figure 4(c). It presents a case where the position of the dotted line is modified, altering the criterion used to decide which triangles we have to refine. In these cases, a slightly different process is applied to correct the appropriate triangles. This algorithm checks each triangle to see whether, with the new criterion, their parent triangle would need a different tessellation. For example, triangles with *I* number 1 or number 10 would not require any change as their parent would experience the same tessellation (or refinement) with both positions of the dotted line. Nevertheless, the parent of the triangle with *I* value equal to 18 had two legs below the dotted line and now both of them are above this line. In this case, the algorithm would coarse the triangle and refine it again. Similarly, triangle 19 (sibling of triangle 18) would also detect that its parent would have been affected by the criterion change.

Following with triangles 18 and 19, we would coarse them eliminating one of them, while the other becomes triangle 4 again. Then, we apply the adequate pattern to refine again the triangle. Similarly, triangles 14, 15, 16, and 17 are affected and three of them are eliminated, while the remaining one becomes triangle 3 and is refined again, creating new triangles with *I* values equal to 14 and 15. These coarsening and refining processes are performed following the methods presented above.

It is important to underline that both processes (coarsening and refining again) are executed at the same time, so that we can coarsen the triangle in more than one level of detail and refine it again. The *I* values have been calculated so that we can know at any point if the triangle we are processing was useful in any of the previous levels of detail. Moreover, although this process seems tedious, only a small portion of the triangles in the mesh will go through this process.

## 4. Ocean Simulation

The previous technique is capable of modifying the details of the mesh in real time to offer a fast rendering of the ocean. It is important to mention that the geometry obtained in this pass will be output and stored in GPU memory, so that it can be used in the following frames for further tessellations or for maintaining the current tessellation if necessary.

Nevertheless, in addition to the geometry management of the mesh simulating the ocean, we must perform other tasks in order to obtain a visually satisfying ocean simulation. In this section we will briefly describe the different techniques used to enhance the realism of the simulation.

### 4.1. Animating Ocean Waves

One of the first features that we must consider is the algorithm applied to simulate the ocean waves. In the state-of-the-art we have presented many techniques that have been developed to model ocean surfaces. Among them, we have selected the Perlin noise [4], as it has been used in many applications and its implementation in current hardware is available [45, 46]. Moreover, Perlin noise is faster than other methods and it is easily ported to GPU shaders, in contrast to other algorithms like the FFT ones which are slower due to the complex calculation process, as previous informal studies showed [47]. In addition, coding the FFT-based animation on the GPU is also quite difficult, as it is necessary to perform several GPU passes to update the texture containing the heightfield at each frame [27, 48]. Other authors proposed precalculating several displacement maps to combine them [10] or select the most suitable one according to the animation time [37]. Nevertheless, in the results section, we have performed a comparison of both animation schemes to analyze their performance within our ocean simulation framework.

In our implementation, the shader in charge of updating the Perlin-based animation of the ocean will calculate the appropriate height according to the position of the vertex within the mesh and to the time of the animation. For enabling the Perlin noise calculation on GPU, we initially upload a texture containing some noise information that is necessary for the real-time noise update.

### 4.2. Rendering Enhancements

In addition to animating the waves, we must also consider other interactions of the ocean, such as refraction, reflection, and foam. Reflection can be obtained by applying environmental mapping on GPU. This technique consists in using 3D texture coordinates to access a cubemap storing the precalculated reflex. The Fresnel term is commonly computed by calculating, for each pixel, the dot product between the normal and the eye vector. This value is used to access a one-dimensional texture which stores different reflections for different fresnel values [49]. In the simulation that we prepared, we only considered reflection and fresnel factor, although we could apply any of the techniques that are available in the literature. These two effects are simple to code and sufficient to offer a realistic impression.

## 5. Results

To analyze the performance and visual results of our ocean simulation, we integrated our approach in an application which controls the tessellation and the final rendering quality. This application was programmed with GLSL and C++ on Windows 7, and the results included in this section have been obtained with a Pentium D 2.8 GHz. with 2 GB. RAM and an nVidia GeForce 8800 GT graphics card.

Before describing the results obtained, it is worth noting that in these tests the distance to the camera has been used as the criterion to guide the tessellation process, indicating which areas need more details. Moreover, screenspace error has been used to limit the tessellation, so that triangles smaller than an indicated size are not further tessellated.

**Figure 7:**
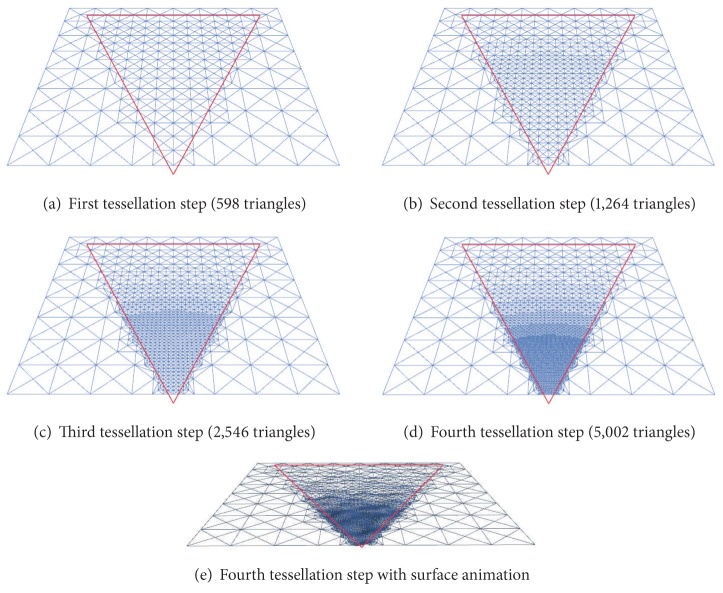
Sample tessellation guided by a simulated frustum (in red).

As an example of how this application works, Figure 7 presents a tessellation example where an initial mesh composed of 256 triangles is refined. In this example, the view frustum of the user (depicted in red) is also used as a tessellation criterion. Then, considering both the frustum and the distance to the viewer, the application decides which areas of the ocean surface to tessellate. Consequently, in the more refined meshes that this Figure presents, it is possible to see how the tessellation is not uniform, as those areas of the mesh which are closer to the observer are more tessellated than those that are farther. In addition, the last image of this figure presents the most detailed surface animated with Perlin noise, where only those triangles within the frustum are animated.

### 5.1. Performance Analysis

To test the performance of our ocean simulation, we present a study of the temporal cost of the whole system. Nevertheless, we would like to start by analyzing why we have decided to use Perlin noise to animate the water surface. In order to test the performance of this displacement technique, we have compared Perlin noise and FFT.

Table 2 offers the performance obtained with our solution when animating ocean meshes of different complexities with both approaches. These results show how the FFT calculations are costlier, as each frame requires two initial rendering passes to prepare two textures as well as some complex calculations when actually adjusting the height of each vertex of the ocean mesh.

**Figure 8:**
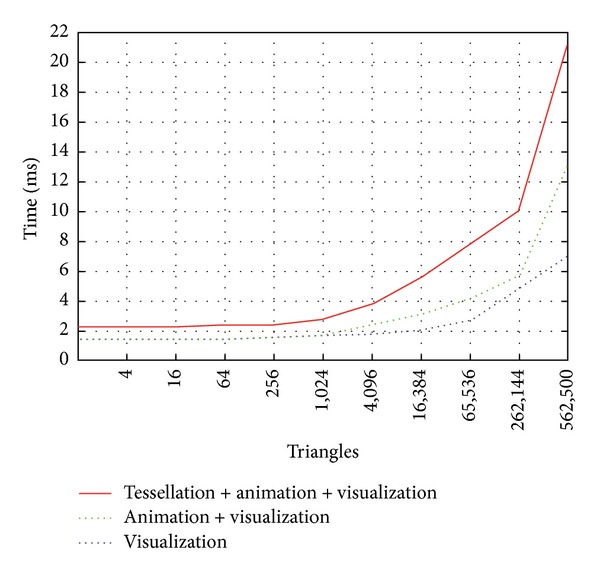
Performance obtained when completely tessellating the mesh.

Figure 8 analyzes the performance of our solution when rendering, animating, and tessellating the surface mesh. In this test, the polygonal complexity of the mesh is equal to 2^*n*^ triangles, being *n* the tessellation step. From the results obtained we can conclude that rendering the ocean mesh is very fast, as although including complex visual effects we can obtain 140 FPS when visualizing more than 500,000 triangles. Updating the animation of the ocean surface entails, in the more detailed approximations, increasing the temporal cost in 70%, while the tessellation represents, on average, an increment of 60%. We must note that, while animating the surface is compulsory for maintaining the visual impression, the application does not require tessellating the surface at each frame. Moreover, in this test, we have considered a very large number of triangles that, in a real application, will not be necessary. We must remember that our tessellation algorithm is view dependent, so that only those areas of the mesh that requires detail area are tessellated (as seen in Figure 7). The reader can find a video showing how the view-dependent tessellation works depending on the field-of-view. In addition, the video also includes the animation of the ocean (see Supplementary video in supplementary material available online at http://dx.doi.org/10.1155/2014/979418).

#### 5.1.1. Performance Comparison

We consider it interesting to compare our solution against the two previously existing ocean simulation frameworks presented by Kryachko [10] and Bruneton et al. [48]. In order to make a fair comparison, the different solutions have been modified so that those features that cannot be compared (e.g., the BRDF calculations of [48]) have been eliminated. In this sense, the test consists in animating a scene with a similar amount of triangles and includes fresnel and some lighting calculations.

The results in Table 3 prove how the performance obtained with our framework is higher. Thus, we can conclude that the implementation of the Perlin noise and the lighting calculations presented above are adequate. Nevertheless, in this test, we have not been able to compare the tessellation as the other approaches do not consider geometry management. Finally, it is worth mentioning that our framework could be easily extended to manage advanced effects as those presented in [48].

### 5.2. Ocean Simulation

**Figure 9:**
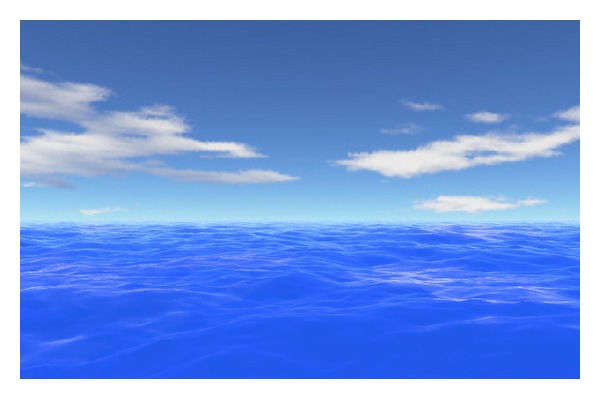
Simulation integrated into the final application.

Finally, Figure 9 presents a snapshot of the ocean simulation that we have proposed. As we mentioned before, the system includes reflections and the fresnel factor to give realism to the scene.

## 6. Conclusions

Ocean simulation has been addressed by many researchers to offer realistic visualization, although some of them were not aimed for real-time animation. In this sense, we have reviewed many related papers in order to choose the main features that affect the realism of the surface of the sea, although only some of them have proposed improvements on the management of the underlying geometry.

We have presented a method for simulating ocean in real time. The presented approach is based on the use of a new adaptive tessellation scheme which exploits coherence among extracted approximations. Accordingly, by storing some information, we are capable of reusing the latest extracted mesh when refining and coarsening the surface. In this framework, the final simulation includes reflection and considers the fresnel term to offer realistic approximations, although our main objective was the development of a new tessellation scheme.

For future work we are focused on the inclusion of more effects like refraction or the interaction of objects with the surface. In this sense, we must perform further research to combine the use of fractal noise with the interactions of objects with the ocean.

From a different perspective, it is worth mentioning that this tessellation algorithm could be also applied to terrain rendering. In this sense, there have previously been ocean techniques applied to terrain rendering, like the projected grid method [30] which was later applied for efficiently visualizing terrain [50]. Moreover, there have also been terrain solutions applied to ocean rendering, like the ocean method presented by Yang et al. [32] which was based on a previously existing GPU-based terrain solution [51]. As a consequence, it is our interest to analyze the possibilities offered by our GPU-based tessellation technique to terrain visualization.

Moreover, the latest graphics API from Microsoft (Direct3D 11) suppose, among other features, the establishment of tessellation as a compulsory feature in real-time applications [52]. This feature could be directly used for view-dependent tessellations of ocean surfaces and, thus, we believe that this unit will be key in the future. We would like to test the performance of our approach moving the tessellation task, now performed in the geometry shader, to the Tessellation units while maintaining the operations of culling and discarding geometry in the geometry shaders.

## Supplementary Material

The reader can find a video showing how the view-dependent tessellation works depending on the field of view. This video also includes the animation of the ocean.

## Figures and Tables

**Algorithm 1:**
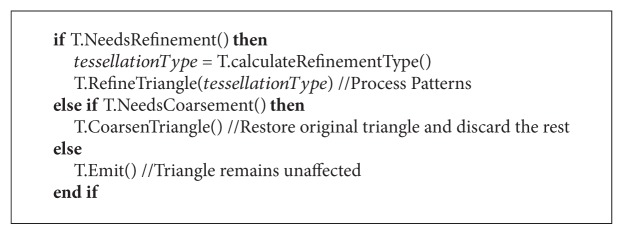
Pseudocode of the main geometry shader.

**Table 1:** Characterization of ocean models.

Authors	GPU usage	Tessellation	Animation technique	Reflection/refraction	Others
Tessendorf [25]	Vertex, pixel	No	Spectral	Yes/yes	Fresnel, caustics, Godrays
Schneider and Westermann [31]	Vertex	Yes	Fractals (Perlin)	Yes/yes	Fresnel
Hinsinger et al. [38]	Vertex buffers	Yes	Parametrical (Gerstner)	Yes/no	Avoid unnecessary animation, fresnel
Johanson [30]	Vertex, pixel	Yes	Fractals (Perlin)	Yes/yes	Fresnel, sunlight
Mitchell [27]	Vertex, pixel	No	Spectral (FFT)	Yes/no	Interactions with objects, wedge
Demers [39]	Vertex, pixel	Yes	Parametrical (Gerstner)	Yes/no	Fresnel, foam, physics
Yang et al. [32]	Vertex, pixel	Yes	Fractal (Perlin)	No/no	Shallow water
Chiu and Chang [40]	Vertex, pixel	—	Spectral (Tessendorf)	Yes/yes	Fresnel, spray dynamics, depth-dependent water color
Fréchot [34]	—	No	Hybrid	No/no	
Hu et al. [37]	Vertex, pixel	Yes	Spectral (Tessendorf)	Yes/yes	Fresnel
Darles et al. [35]	—	Yes	Hybrid (Tessendorf)	Yes/yes	Fresnel, glare, foam
Thürey et al. [22]	—	No	Physical (Navier-Stokes)	Yes/no	Shallow water, foam, interactions with objects,
Bruneton et al. [48]	Vertex, pixel geometry	No	Parametrical (Gerstner)	Yes/yes	BRDF, fresnel, wedge
Our proposal	Vertex, pixel geometry	Yes	Fractals (Perlin)	Yes/no	Fresnel

**Table 2:** Comparison of time (in milliseconds) required for animating ocean surfaces with different polygonal complexities with Perlin noise and a FFT-based solution.

Number of triangles	Perlin noise	FFT
4,096	2.45	4.89
16,384	3.14	6.45
65,536	4.18	7.22
262,144	5.69	10.23
562,500	13.25	18.65

**Table 3:** Comparison of time (in milliseconds) required for visualizing ocean surfaces with similar polygonal complexities using different solutions.

Solution	Number of triangles	FPS
Kryachko [10]	293,470	139
Bruneton et al. [48]	226,200	137
Our solution	250,000	225
